# Determinants of Fertility Intentions of the Women in Bosnia and Herzegovina—An Example from the Semberija Region

**DOI:** 10.3390/bs13050417

**Published:** 2023-05-15

**Authors:** Rada Golub, Andjelija Ivkov-Dzigurski, Vlado Simeunović

**Affiliations:** 1 Faculty of Education, University of East Sarajevo, 71126 Lukavica, Bosnia and Herzegovina; 2 Department of Geography, Tourism and Hotel Management, Faculty of Sciences, University of Novi Sad, 21000 Novi Sad, Serbia

**Keywords:** fertility, socio-demographic factors, Semberija

## Abstract

The aim of this paper was to analyze the socio-demographic determinants of fertility in Bosnia and Herzegovina that affect the number of live births in Semberija. The paper focuses on work and educational statuses, economic crisis and unemployment, and other relevant factors that affect desired family sizes and lead to negative demographic trends. The relevant data were collected through a survey questionnaire filled by a verified sample of 1000 women in their reproductive period (aged 15–49). In order to evaluate the role of each research variable in predicting fertility intentions, the arithmetic mean, frequency of responses in percentage, Pearson’s coefficient, and a logistic regression model were used to explore the related factors of fertility behaviors among women in this population. The results showed that employment status, poor housing conditions, and financial support from the state statistically significantly impact future birth trends. Those socio-demographic factors affect desired family sizes and have proven essential to future fertile behavior.

## 1. Introduction

The fertility rate results from several factors whose understanding is critical for solving the problem of fertility rates below replacement levels. Birth and fertility rates are affected by numerous biological, economic, social, and psychological factors. This paper places special emphasis on socio-demographic factors that largely determine childbearing and desired family sizes. The latest demographic trends in developed nations indicate a growing tendency for women, men, and couples to delay having children until later in life. This has resulted in a considerable proportion of the population having fewer children than they desire or remaining childless [1,2].

One of the primary factors that decreases the desire for fertility is the socio-economic transformation of society, which results in greater anticipated expenses associated with raising a greater number of children. The main driving force that reduces desired fertility is the structural socio-economic transformation of society, which leads to increased expected costs of having a larger number of children. A larger number of offspring requires higher costs, which places economic domination in the context of the decisive factor for expanding the family in accordance with rational and conscious decisions of couples about childbirth [3]. The number of children parents would have if there were no personal or economic barriers regulating fertility represents the desired family size [4].

On the other hand, when structural socio-economic transformations slow down, as is the case in developed countries, desired fertility rates increase. Of course, this depends on multiple factors, including demographic, cultural, and social factors [5]. 

In high-income societies, having a permanent job and a stable income is a key requirement for many people before considering having children. Uncertainty about economic and financial security often leads individuals to postpone childbearing [6,7,8,9].

Occupations, income, and poor housing conditions have triggered a number of changes, not only in the size of a family, but also in its survival. High costs of preschool institutions, non-flexible working hours for new mothers, political and economic situation, and unemployment are some factors determining aspects of family life. Contemporary European research has shown that a decline in fertility and population aging will result from changes in the societies’ value systems, housing conditions, and economic uncertainty [10,11]. According to the findings [12], social change, the political situation, and economic growth and decline are all reflected in the demographic picture of a country. Finally, and more importantly, it has been proved that employment insecurity and financial uncertainty delay family formation [13,14].

According to the literature, these trends are related to socio-demographic changes such as urbanization, a general increase in levels of education, and women’s increased labor force participation, suggesting that the fertility rate could fall even lower as these processes persist in the future [15]. The economic crisis and uncertainty that affected Europe and the US also impacted fertility rates and the real economy. Economic shocks dramatically affected family dynamics, with 22 out of 32 Western countries experiencing a decline in fertility rates between 2008 and 2013. The worsening of the labor market during the Great Recession was largely responsible for the negative effects on fertility rates. The sudden increase in unemployment rates during the recession reduced the total fertility rate in the West by 3% since the beginning of the crisis [16,17]. Permanently employed women have a higher number of children than those in insecure employment or those searching for work. Employed mothers opt for more flexible working arrangements with lower incomes to dedicate more time to their family life. In countries with high unemployment rates and among women holding temporary contracts, there is a discernible delay in the decision to have a second child. Uncertainties in the labor market will lead to women delaying or revising their reproductive plans [18,19]. Recent research indicates that in wealthy countries over the past decade, fertility rates may be impacted not only by unemployment or income levels, but also by perceived economic and financial uncertainty, leading to a delay or abandonment of childbearing [6,8,9]. This means that the perceptions of insecurity regarding welfare state provisions may have a greater impact on current plans for having children than the individual’s employment situation or government policies on families. For instance, childbearing plans may be more affected by perceptions of insecurity related to welfare state provisions than the direct employment situation or family policy legislation [6,20].

A higher level of education implies a longer study period, resulting in negative effects on the marriage market with the tendency to delay the first childbearing and shorten the fertility period. There has been a significant decline in the total fertility rate (TFR) among women with higher levels of education [21]. As Rašević and Vasić [22] present, level of education is one of the most important indicators of social status. Societies with unstable socio-economic conditions, a high degree of economic insecurity, and insufficiently branched measures for reconciling work and parenthood have a negative relationship between education and fertility [23].

Semberija is located in the very northeast of Bosnia and Herzegovina, and although it has a favorable geographical position and a high population density of 141.4 inhabitants per km^2^, its basic demographic trait is non-parturition. The existing dynamics of achieved fertility are adapted to the modern reproductive model, i.e., insufficient number of births. Nowadays, the total fertility rate in Semberija is 1.4 (2020), which is not enough for simple reproduction—a level of 2.1 children per woman—implying that women in Semberija do not give birth to their reproductive replacements. 

Considering this regional unit’s fertility characteristics, the effects of socio-demographic factors on fertility rates were examined. The aim is to determine how these factors affect the desired family size.

## 2. Materials and Methods

For the purposes of this research, a survey questionnaire was conducted on a stratified sample of 1000 mothers in their reproductive period (aged 15–49). The stratification was performed by age (15–49), employment status (employed, unemployed, and temporarily employed), and education status (primary, secondary, tertiary). The survey was conducted in March 2018, and 357 women from rural, 575 women from urban, and 68 women from suburban areas took part. The questionnaire was obtained from the CDR (Center for Demographic Research) and used for the purposes of a doctoral dissertation. The questionnaire consisted of 20 questions on socio-demographic determinants of fertility, divided into three groups. The first group of questions referred to general demographic characteristics of women (age, place of residence, marital status, health issues), the second group of questions was based on economic characteristics of families (financial and employment status, education, occupation, housing, balance between work and parenting), and the third group of questions referred to possible population policy measures. The total number of both planned and correctly completed questionnaires was 1000, which represents 4.3% of the total fertile female population in Semberija. 

The statistical analysis included examination of socio-demographic determinants such as education and employment statuses, economic crisis and unemployment, lack of financial support from the state, high childcare costs in preschool institutions, mismatch between work and parenting, delayed marriage, and other relevant factors affecting fertility changes in Semberija. Based on the respondents’ attitudes, we will explain the significance of the mentioned variables in shaping the modern reproduction regime. The statistical program IMB SPSS-19 was used for data entry and analysis, descriptive statistics, and statistical inference. We used Pearson’s correlation to determine if there was a connection between desired and achieved reproduction. 

In order to examine the influence of socio-demographic variables on birth prediction, we used a binary logistic regression. Using logistic regression, also referred to as a logistic model or logit model, the logistic curve is applied to the data in order to predict the likelihood of an event. Logistic regression is a type of regression analysis where the dependent (criterion) variable is dichotomous, i.e., binary, and is coded as 0 or 1, and there is at least one independent (predictor) variable. As events in the social sciences are often dichotomous, logistic regression is often used in predicting these events.

This is a logistic function, which is often called a sigmoid function. It is, by its nature, derivable in the entire domain and is defined as in Equation (1):

(1) $$\sigma \left(\alpha \right)=\frac{1}{1+exp\left(-\alpha \right)}$$

The logistic regression model can be represented as follows in Equation (2):

(2) $$h\;\left(x,w\right)=\sigma \left(w^{T\;}\emptyset \;\left(x\right)\right)=\frac{1}{1+exp-w^{T}\emptyset \left(x\right))}=P(y=1/x)$$

To make it easier to understand whether a woman from the sample will decide to give birth, we used a probability form as in Equation (3).

(3) $$p=\frac{exp\left(b_{0}+\sum_{j=1}^{m}b_{j}x_{j}\right)}{1+exp\left(b_{0}+\sum_{j=1}^{m}b_{j}x_{j}\right)}$$

Logistic regression uses several methods that are applied depending on the research problem. In this paper, we used the ENTER method.

### 2.1. Dependent Variable

Our sample included 1000 respondents. We did not have any respondents who were not included. The independent variable was represented by the data on “continuation of childbearing”: 0 = not planning to give birth, and 1 = planning to give birth. Based on the total sample, we had 612 women who plan to give birth and 388 women who do not plan to give birth.

### 2.2. Independent Variable

The set of predictors included a total of 17 variables, namely: respondent’s education, type of settlement in which they live, average age of birth, size of the apartment where the family lives, employment status, place of employment, financial status of the family, economic status of the family, poor housing conditions, lack of financial support from the state, later-in-life marriage, higher employment, insufficient number of places for children in preschool institutions, high cost of preschool institutions, mismatch between work and parenting, uncertain future, and shortened maternity leave.

## 3. Results

In accordance with the aim of the research, only female respondents in their fertile period (aged 15–49) participated in the survey. Table 1 shows the frequency of their responses related to the desired and actual number of children, employment and education statuses, and the average number of children they gave birth to. 

Based on the descriptive statistics shown in Table 1 and using IBM SPSS Statistics (version 26.0, IBM Corp., Armonk, NY, USA), the significance of the mentioned parameters to the fertility rate in Semberija was examined.

According to the attitudes of the respondents, differences between the desired and the actual number of children can be noticed. A total of 90.5% of mothers who desired one child achieved their desired number of children; 68.1% referred to mothers who desired two children, while the percentage decreased significantly as the desired number of children increased. Thus, the percentage was the smallest when the desired number of children was three or four, since this was achieved by only 31.4% or 31.3% of the respondents (Table 1). 

According to the structure of the answers, Pearson’s correlation was applied in order to determine if there was a connection between desired and achieved reproduction. The interviewees expressed a greater desire to have children the fewer children they already had, as evidenced by Pearson’s coefficient (0.523), which highlights the strong correlation between the two variables. The average number of children the respondents gave birth to was 1.96, and the desired number was 2.53. Most respondents (483) stated that they wanted two, and 379 wanted three children.

When asked if the mentioned measures existed and if they enabled them to give birth to the desired number of children, 61.2% of the respondents stated that they would give birth, and 38.8% of them did not want more children, regardless of the measures.Block zero does not represent a significant process, but it shows us how to predict future births without introducing predictor variables. In a mathematical sense, it is the quotient between the number of women who want to give birth and those who will not give birth again. Exp (b) cannot be negative; its value indicates the direction of connection:

1. Exp (b) from 0 to 1—Negative correlation
2. Exp (b) = 1—No correlation
3. Exp (b) > 1—Positive correlation) [24]

In our case, it is 1.577, which indicates a positive correlation; that is, there is the possibility that women will decide to have the desired number of children if existential issues are resolved (Table 2).

The Omnibus Test is used for testing a hypothesis. We tested the hypothesis that the logistic coefficients for all predictor variables equal zero as in Equation (4).

H_0_ = β_1_ = β_1_… β_m_ = 0 (4)

The Omnibus Tests of Model Coefficients table shows the summary indicators of model performance. That test is called goodness of fit and shows how accurately the model predicts the results. Of course, this set of results should be as significant as possible; that is, the value of Sig. should be less than 0.05. In this case, the significance is 0.00, i.e., *p* < 0.05, from which it can be concluded that this model predicts the data well [14].

Table 3 shows that our chi-square is 83,619, and with seventeen degrees of freedom, the number of predictor variables is statistically significant (*p* = 0.000). Based on that, we reject the hypothesis that all logistic coefficients in the population are equal to zero and have no partial contribution.

The −2 log-likelihood is similar to the sum of squared residuals in a linear regression. It needs to be as small as possible. In the Model Summary table, you can see the values of the Cox and Snell R Square and Nagelkerke R Square, which show how much of the dependent variable is explained by the model. In this case, for the final model, those values are 0.080 and 0.109. In other words, the set of variables that make up the obtained model explains between 8% and 10.9% of the variance (Table 4).

Model evaluation indicates the percentage of correct classifications based on the model, and the closer the value is to 100%, the better the model. It is an indicator of the predictive value of the logistic model. It is analogous to the coefficient of determination and standard error in linear regression.

We see that this model of future birth gives 66.8% correct classifications (Table 5).

That is 16.8% more than if we randomly classified women, because when the predictor is a binary variable, the probability of a random guess is 50% (66.8 − 50 = 16.8%).

The first step (Table 6) shows us the variables in the logistic equation. The table also indicates a parameter that shows us the evaluation of the probability parameters of the logarithm of the chances that show if a woman will decide to give birth in the future.

Here, we can test the null hypothesis for the partial contribution of the predictor variables to the overall probability of predicting the so-called “continuation of childbearing”. This result can be obtained by the Wald statistic [25]. It can be calculated as W=$\frac{B}{\;SE}$ (logistic coefficient through standard error for a particular coefficient).

In the significance column, we can see that the following variables statistically influence future births: employment status, poor housing conditions, and lack of financial assistance from the state. This does not mean that other variables do not influence the decision on future childbearing, but it means that these three variables have a particular predictive contribution to the decision on future childbearing.

Looking at the set of variables that are statistically significant, an answer to the question about further childbearing is logically imposed because if a person does not have a safe and well-paid job, nor do they have an adequate apartment, and if there is no systemic support, they will not give birth in the future, and vice versa.

Obviously, financial security here is emphasized as a motive immediately above the primary motives.

The interpretation of the exponentiated logistic coefficient (Exp(B)) should be emphasized in the table. This is an exponentiated quotient of the chance of the target category occurring if we increase it by one compared to the initial state.

Looking at the Table 6, we see that, for example, it is 1.350 times more likely that a woman will choose to give birth if she is employed, it is also 1.388 times more likely she will do it if housing conditions are better, and it is 1.208 times more likely she will give birth in future if there are systemic measures of the state aimed to support a family that decides to have more children.

## 4. Discussion

The research results suggest that the factors that significantly influenced family transformation and caused changes in fertility in Bosnia and Herzegovina are attributed to socio-economic determinants. The research proves that a mismatch between the desired and actual number of children exists. The desired number of children does not match the achieved average. The fact that 61.2% out of 1000 respondents would give birth in the future is encouraging, and that would provide significant results regarding the required fertility at the local and regional levels. Even though 38.8% of the respondents declared that they did not want to have more children, those respondents generally achieved the desired number of children, i.e., they gave birth to 2.35 children on average. Achieving the desired number of children is crucial for completing the demographic picture of Semberija, and it would contribute to moving the current total fertility rate from its critical point. It has been discovered that fertility intentions and demographic, socio-economic, and cultural standards can predict actual birth rates. The decision to have more children and the IPI (interpregnancy interval) are influenced by micro-level variables such as socio-economic level, education, employment, a feeling of empowerment, age, and household responsibilities [26].

According to research conducted in Hong Kong using logistic regressions, the factors that influence low fertility intentions differ depending on the parity: satisfaction with marital life, income in the household, and good husband-wife communication are all positively correlated with first-birth intentions; wives’ part-time employment lowers second-birth intentions; wives’ full-time employment and inequalities between genders in the distribution of household duties are inversely linked to third-birth intentions. It is important to state that, independent of other socio-economic determinants, fertility desire has developed into a powerful indicator of fertility intention, particularly with regard to first and second births. Actual parities also differ with regard to the reasons for having children and the challenges of raising them [27]. 

Concern for the future of their child is the most common reason parents decide not to have children. Employment status and income, as well as lack of housing, triggered a number of changes, not only in family size, but also in its survival. Since a family is considered a consumer community nowadays, a larger number of children represents quite a challenge and financial burden for parents. Modern educational and behavioral patterns, everyday acts, and socializing dictate financial challenges to future parents that are considered obstacles to childbearing [28]. The non-existential basis of a child’s value shapes the modern regime of reproduction, i.e., the birth of fewer children (one or two) or none at all.

Based on the attitudes of the respondents, employment was quoted as the most determining factor of fertility. Unemployed women have, on average, given birth to 1.9 children, employed women have given birth to an average of 2.1 children, and temporarily employed women have had 1.73 children on average. Unemployment as a fundamental trait of an uncertain future and economic crisis indirectly results in delayed marriages and delayed childbearing. Over the last few decades, the participation of women, including those who are mothers, in the labor market has increased, even though women are much more involved in childrearing than men, so due to incompatibilities between their roles in the labor market and in the family, this “double shift” can put pressure on many working mothers [29,30]. Compared to stay-at-home mothers, working women were much more likely to have positive fertility goals [30]. Research in Europe also shows that the additionally desired family size is adversely correlated with age (for all three parities) and being inactive (unemployed) at the person level [31]. Exploratory factor analyses provided evidence for a five-factor model for the subscale measuring negative motives for having children (i.e., the weight of and inexperience in childrearing, social and ecological anxiety, marital stress, financial difficulties and economic limits, and physical discomfort and self-image worries) [32]. Therefore, it is justified that permanently employed women have more children (2.1) than those with insecure employment (1.9) or those in search of one (1.7). Working mothers decide on more flexible jobs with lower wages in order to dedicate their free time to their families. This is also confirmed by the obtained research results, which show that, on average, employed mothers gave birth to more children.

Based on the attitudes, the lack of financial support from the state and municipality proved to be another effective factor in parenting; 72.9% of the respondents who gave birth (on average to 1.95 children) agree with this statement, which is far below the desired number. Countries might set up institutions to reduce some incompatibilities between employment and childrearing (secure employment, flexible working hours) [33]. The Japanese government has taken numerous steps to combat the country’s dropping birthrate, including expanding daycare services, changing the law regarding parental leave, and promoting a strict work-life balance policy. These initiatives, if anything, are directed at white-collar workers [34]. The economic component is still the key factor for getting married early, survival of the family, and meeting basic life needs. Another result of the research shows that fertility intention is related to the perceived cost of having children. The results of the logistic regression also suggest that people’s intention to have children in the future increases when the mean score of perceived childbearing costs decreases. The findings from research conducted in Turkey revealed a substantial correlation between individuals’ intentions to have children and the costs associated with having children. Policymakers can alter the cost–benefit ratio of having big and small families by knowing children’s values and expenses [35,36,37].

The size of the family is conditioned by the size of the living space. There is a legally regulated solution in developed countries around the world, where the necessary size of the living space is prescribed according to the number of family members, and as a pronatalist incentive measure, state aid is provided for the necessary purchase. Poor housing conditions proved to be a very important factor in predicting future childbearing. The number of square meters of living space should increase with the number of children. However, with urbanization, families have adapted to modern living conditions—smaller apartments and fewer family members. The research results have shown that smaller housing units (below 50 m^2^) supported fewer family members, i.e., an average of 1.67 children, while those above that square footage had 2.04 children.

Studies of socio-demographic determinants of fertility have yielded similar results all over Europe. Financial factors have been cited as the most important factors of fertility in numerous studies [21,24,25,27]. The tendency is downward, according to all articles assessing fertility status in the context of economic restrictions. There is some evidence that the countries hardest hit by the recent Great Recession faced declines in fertility, particularly among young people. Economic hardships increase the probability of unemployment or temporary employment, leading to delaying family formation [38]. Additionally, one of the worst effects of the economic crisis is employment instability, which has a significant impact on giving birth to more children [7]. A study conducted in Serbia [39] explains that 71% of female respondents over 30 believed that a great economic crisis occurred, which had a decisive impact on postponing childbirth. Many variables correlated with income impact fertility, such as education, occupation, housing location, potential spouse income, lifestyle habits, costs, and benefits of raising children. Having dual incomes, that is, both spouses are employed, will enable better conditions for family formation. With increased income, the psychological stress of having children decreases. Parental care for the existence, adequate schooling, children’s interests, and of course, the possibilities of their care while busy with work obligations significantly impact decisions about childbirth. With an increase in income and better material status, tendencies to expand the family also increase. Among women who believe there is a significant difference between potential and achieved fertility, economic restrictions seem to be the leading cause of limited fertility [18].

The economic cost of raising children is a significant obstacle to evaluating and actualizing reproductive norms. Financial status is an important determinant in meeting human needs [40]. In order to stimulate the childbearing processes, various financial and other kinds of support should be provided in order to encourage marriages, reduce the number of divorces, and enable participation of women in the labor market as well as their return to work after maternity leave, which should be stimulated by flexible working hours, alternative care, and education [41].

Socio-demographic determinants of fertility in the Republic of Srpska (an entity of Bosnia and Herzegovina) were presented in the scientific research project “The analysis of demographic situation and implementation of pronatal measures and activities in the Republic of Srpska” in 2009. According to the research, in the case of future ideal economic, political, and health conditions, 68.5% of women in the Republic of Srpska would give birth, while in the region of Semberija, the percentage of women willing to give birth in the same circumstances is only 40%. Furthermore, significant deviations in fertility were observed in connection to women’s level of education.

Future social and demographic policies ought to consider these changing trends in population demographic behavior and attempt to envision a number of actions that should address fertility and family issues in a sustainable, collaborative way, conveyed within a regional as well as a global scope [42]. Overall, the findings show that fertility intention is not a simple idea. Instead, it entails a complicated fertility choice-making procedure. The impacts of multiple factors on the optimal family size highlight the link between fertility intention and the complex procedures of fertility decision-making. Therefore, using a single indicator as its proxy variable (such as the ideal number of children) is inappropriate. The causes of this situation should also be sought in other decisive factors that influence the formation of certain value attitudes and decision-making. For example, employment does not necessarily affect economic stability, but rather the level of income and solved housing issues [28].

According to the effect sizes of specific factors, it is essential to develop appropriate fertility promotion plans for families at different phases of fertility [43]. In order to slow down the negative demographic trends, certain measures should be implemented immediately, starting with the reform of the socio-economic system [44]. Nevertheless, the findings of this study indicate that in the future, poor housing conditions, employment stabilization, and lack of financial support from the state and municipality (which would reduce the direct costs of raising children) should be prioritized over other measures [34,45]. The local government should take into account the low employment rate and implement its increase in the development strategy as one of the future development goals of the city and the region.

## 5. Conclusions

Semberija faces a decline in fertility due to the decline in the absolute number of live births, postponement of the first birth, and delayed marriages in line with other socio-demographic factors recognized as structural barriers in realizing and shaping the desired family size. 

This research is dedicated to the determinants of decreased fertility in Semberija, with the aim to identify the community context in this region that shapes both the actual and desired size of the family, as well as to identify possible target groups for future family planning programs. The survey results confirm that the aforementioned determinants, such as employment status, poor housing conditions, and state financial support, strongly impact decisions regarding the desired number of children. The achieved fertility of employed mothers is higher compared to mothers with unstable employment, and it ranges below two children per woman. The local government should take into account the low employment rate and implement its increase in the development strategy as one of the future development goals of the city and the region. The attitude of most surveyed mothers is that 2.53 represents their desired number of children in conditions with no structural obstacles. That would also satisfy the parameters of simple reproduction. Based on the research results, it can be concluded that difficulties in achieving desired family size exist and that socio-demographic determinants strongly affect the births of the desired number of children. Therefore, it is necessary to provide support to the family through state, local, and regional institutions. The most often mentioned form of state aid is the so-called “financial injection” as the leading stimulus for childbearing. However, this should imply the provision of not only short-term assistance at a given moment, but also financial inflow through solving unemployment, subsidies for home loans for large families, and other financial benefits that will reduce family expenses.

Based on the offered results, it can be concluded that the offered model can be used to predict the “continuation of births” of the examined group of women in Bosnia and Herzegovina.

It would make sense to include other variables, such as personality traits, in this model because they can significantly determine attitudes toward childbirth in general. The results of this research should be used for the ultimate goal, which is population growth in the future.

The only solution to overcoming the problem of low fertility is to introduce stronger and more radical measures of population policy in the coming period. Nowadays, Semberija should adopt and follow an agenda that can strengthen national identity through socio-economic measures, as they represent key factors that shape negative trends in this region.

## Figures and Tables

**Table 1:** Socio-demographic indicators of respondents.

Variable	Question	Frequency	Average Birth
Number of children	Actual number of children	100.0	1.9
	Desired number of children	100.0	2.5
Average birth age	15–19 20–24 25–29 30–34 35–39 40+	8.4 34.3 34.9 17.8 3.9 0.7	2.2 2.13 1.91 1.75 1.44 1.57
Type of settlement	Village City Suburbs	35.7 57.5 6.8	2.17 1.84 1.9
Education	Primary	6.8	2.4
	Secondary	52.6	2.0
	Tertiary	40.6	1.8
Employment	Employed	40.1	2.1
	Unemployed Temporary employed	46.1 13.8	1.9 1.7
Place of employment	Own Private State	7.6 27.4 29.0	2.11 1.85 1.88
Apartment surface	0–50 51–100 101–150 over 150	11.2 58.0 12.9 8.4	1.94 1.94 2.02 2.04
Financial status	Very good Good Bad Very bad	10.6 81.0 7.8 0.6	1.9 1.97 1.88 2.33
Economic status	Agreed	78.4	1.96
Poor housing conditions	Agreed	73.5	1.94
Lack of financial support from the state	Agreed	72.9	1.95
Later-in-life marriage	Agreed	60.1	1.94
Higher employment	Agreed	51.2	1.93
Insufficient number of preschool institutions	Agreed	58.0	1.92
High childcare costs in preschool institution	Agreed	55.0	1.98
Mismatch between work and parenting	Agreed	63.7	1.95
Uncertain future	Agreed	71.2	1.97
Shortened maternity leave	Agreed	64.3	1.85
Future birth	Yes	61.2	1.82
	No	38.8	2.35

**Table 2:** Variables in the equation.

		B	S.E.	Wald	df	Sig.	Exp(B)
Step 0	Constant	0.456	0.065	49.317	1	0.000	1.577

**Table 3:** Omnibus Tests of Model Coefficients.

	Chi Square	df	Sig.
Step 1 Block Model	83.618	17	0.000 **
	83.618	17	0.000
	83.618	17	0.000

** Significant at the level *p* < 0.001.

**Table 4:** Model Summary.

	−2 Log-Likelihood	Cox and Snell R Square	Nagelkerke R Square
Step 1	1252.071 ^a^	0.080	0.109

^a^ Positive score st (a,b) means that a and b are more likely to be aligned by evolution than by chance.

**Table 5:** Predicted.

Observed		No	Yes	Percentage Correct
Yes	No	128	260	33.0
Yes		72	540	88.2
Overall Percentage				66.8

**Table 6:** Variables in the Equation.

		B	S.E.	Wald	df	Sig.	Exp(B)	95% C.I. for EXP(B)	
								Lower	Upper
Step 1 ^a^	Education	0.078	0.140	0.309	1	0.578	1.081	0.822	1.421
	Type of settlement	0.035	0.154	0.051	1	0.822	1.035	0.766	1.399
	Average age birth	−0.019	0.074	0.069	1	0.794	0.981	0.848	1.134
	Apartment surface	0.125	0.071	3.073	1	0.080	1.133	0.985	1.303
	Employment status	0.300	0.146	4.238	1	0.040	1.350	1.014	1.797
	Place of employment	−0.066	0.080	0.674	1	0.412	0.936	0.800	1.095
	Financial status	0.162	0.155	1.095	1	0.295	1.176	0.868	1.592
	Economic status	0.115	0.086	1.801	1	0.180	1.122	0.948	1.327
	Poor housing conditions	0.291	0.086	11.456	1	0.001	1.338	1.130	1.583
	The lack of financial support from the state	0.189	0.087	4.761	1	0.029	1.208	1.019	1.433
	Late-in-life marriage	−0.007	0.068	0.011	1	0.917	0.993	0.868	1.136
	Higher employment	−0.019	0.069	0.074	1	0.785	0.981	0.857	1.124
	Insufficient number of preschool institutions	−0.043	0.084	0.257	1	0.612	0.958	0.813	1.130
	High childcare costs in preschool institution	0.045	0.079	0.326	1	0.568	1.046	0.895	1.223
	Mismatch between work and parenting	0.049	0.087	0.325	1	0.569	1.051	0.887	1.245
	Uncertain future	−0.037	0.077	0.234	1	0.629	0.964	0.829	1.120
	Shortened maternity leave	−0.026	0.075	0.119	1	0.730	0.974	0.841	1.129
	Constant	−2.87	0.611	22.195	1	0.000	0.056		

^a^ Variable(s) entered in step 1: education, type of settlements, average age birth, apartment surface, working status, company ownership, financial status, economic status, poor housing conditions, lack of financial support from state, late-in-life marriage, higher employment, insufficient number of preschool institutions, high childcare costs in preschool institutions, mismatch between work and parenting, uncertain future, shortened maternity leave.

## Data Availability

The data presented in this study are openly available in Mendeley data at doi: 10.17632/hkkrvjphhv.1.

## References

[1] Schmidt L., Sobotka T., Bentzen J.G., Andersen A.N., ESHRE Reproduction and Society Task Force (2011). Demographic and medical consequences of the postponement of parenthood. Hum. Reprod. Updat..

[2] Sobotka T., Beaujouan É. (2017). Late Motherhood in Low-Fertility Countries: Reproductive Intentions, Trends and Consequences. Preventing Age Related Fertility Loss.

[3] Muhoza D.N., Broekhuis A., Hooimeijer P. (2014). Variations in Desired Family Size and Excess Fertility in East Africa. Int. J. Popul. Res..

[4] Thomson E. (2015). Family Size Preferences.

[5] Hansen H.T. (2019). Theories of fertility decline and the evidence from development indicators. Popul. Develop. Rev..

[6] Savelieva K., Jokela M., Rotkirchc A. (2023). Population Rese. Reasons to postpone or renounce childbearing during fertility decline in Finland. Marriage Family Rev..

[7] Goldstein J., Örsal D.D.K., Kreyenfeld M., Jasilioniene A. (2013). Fertility Reactions to the “Great Recession” in Europe. Demogr. Res..

[8] Lebano A., Jamieson L. (2020). Childbearing in Italy and Spain: Postponement Narratives. Popul. Dev. Rev..

[9] Brinton M.C., Bueno X., Oláh L., Hellum M. (2018). Postindustrial Fertility Ideals, Intentions, and Gender Inequality: A Comparative Qualitative Analysis. Popul. Dev. Rev..

[10] Söderberg M., Christensson K., Lundgren I., Hildingsson I. (2015). Women’s attitudes towards fertility and childbearing–A study based on a national sample of Swedish women validating the Attitudes to Fertility and Childbearing Scale (AFCS). Sex Reprod. Health.

[11] Jakovljevic M. (2017). Population ageing alongide healt carespending growth. Serb. Arh. Med..

[12] Andre T., Oncea B., Capatana C., Bucerzan I. (2015). Characteristic of the population of Romania during 1990–2013. Transyl-vanian Rev. Adm. Sci..

[13] Caltabiano M., Comolli C.L., Rosina A. (2017). The effect of the Great Recession on permanent childlessness in Italy. Demogr. Res..

[14] Zeman K., Beaujouan E., Brzozowska Z., Sobotka T. (2018). Cohort fertility decline in low fertility countries: Decomposition using parity progression ratios. Demogr. Res..

[15] Coutinho R.Z., Golgher A.B. (2018). Modelling the proximate determinants of fertility for Brazil: The advent of competing preferences. Rev. Bras. Estudos Popuatio.

[16] Comoli C.L. (2017). The fertility response to the Great Recession in Europe and the United States: Structural economic conditions and perceived economic uncertainty. Demogr. Res..

[17] Matysiak A., Sobotka T., Vignoli D. (2020). The Great Recession and Fertility in Europe: A Sub-national Analysis. Eur. J. Popul..

[18] Adsera A. (2011). The interplay of employment uncertainty and education in explaining second births in Europe. Demogr. Res..

[19] Kreyenfeld M., Andersson G. (2014). Socioeconomic differences in the unemployment and fertility nexus: Evidence from Denmark and Germany. Adv. Life Course Res..

[20] Comolli C.L., Neyer G., Andersson G., Dommermuth L., Fallesen P., Jalovaara M., Jónsson A.K., Kolk M., Lappegård T. (2020). Beyond the Economic Gaze: Childbearing During and After Recessions in the Nordic Countries. Eur. J. Popul..

[21] Pobrić A., Sivac A. (2017). The education-fertility relationship in Bosnia and Herzegovina. Geogr. Pregl..

[22] Rašević M., Vasić P. (2017). Obrazovanje kao faktor fertiliteta u Srbiji. Ann. Ser. Hist. Sociol..

[23] Brzozowska Z., Mynarska M. (2017). Fertility intentions and their realization: Insight from the polish generations and gender sur-vey. Work. Pap..

[24] Pešić M. (2016). Upotreba Logisticke Regresije u Modeliranju Verovatnoce Bankrota Preduzeca.

[25] Ward Michael D., Ahlquist John S. (2018). Maximum Likelihood for Social Science: Strategies for Analysis.

[26] Preis H., Tovim S., Mor P., Grisaru-Granovsky S., Samueloff A., Benyamini Y. (2020). Fertility intentions and the way they change following birth-a prospective longitudinal study. BMC Pregnancy Childbirth.

[27] Cheng H., Luo W., Si S., Xin H., Peng Z., Zhou H., Liu H., Yu Y. (2022). Global trends in total fertility rate and its relation to national wealth, life expectancy and female education. Publ. Health.

[28] Marinković M., Perić-Romić R., Petrašević A., Perendija V., Majić A. (2020). Socio-demografska analiza stavova porodilja o plani-ranju porodice I mjerama populacione politike u Republici Srpskoj. Demografija.

[29] Akrap A., Čipin I. (2011). Usklađivanje poslovnoga i obiteljskoga života u Hrvatskoj: Utjecaj na fertilitet. Drustvena Istraz..

[30] Gehad S., Saleh M., Alareed H., El-Shabrawy E., Elbahrawe R. (2022). Effects of sociodemographic background on fertility motivation patterns in the Beni-Suef governorate, Upper Egypt. J. Taibah Univ. Med. Sci..

[31] Testa M.R. (2014). On the positive correlation between education and fertility intentions in Europe: Individual-and country-level evidence. Adv. Life Course Res..

[32] Guedes M., Pereira M., Pires R., Carvalho P., Canavarro M. (2015). Childbearing Motivations Scale: Construction of a New Meas-ure and its Preliminary Psychometric Properties. J. Child Family Stud..

[33] Behrman J., Gonalons-Pons P. (2020). Women’s employment and fertility in a global perspective (1960–2015). Demogr. Res..

[34] Nishimura T. (2012). What Are the Factors of the Gap between Desired and Actual Fertility?—A Comparative Study of Four Devel-Oped Countries.

[35] Kohlman A. (2002). Fertility Intentions in a Cross Cultural View the Value of Childern Reconsiderd.

[36] Vlassoff M. (1982). Economic utility of children and fertility in rural India. Popul. Stud..

[37] Fazeli E., Golmakani N., Taghipour A., Shakeri M. (2014). Relationship between value of children and fertility rate in womem re-ferred to Mashhad helth centers. Iran. J. Obstet. Gynecol. Infertil..

[38] Lange M., Wolbers M.H.J., Gesthuizen M., Ultee W.C. (2014). The Impact of Macro- and Micro-Economic Uncertainty on Family Formation in The Netherlands Marloes de Lange. Eur. J. Popul..

[39] Veljović R. (2015). Demografski Aspekti Fenomena Odlaganja Rađanja u Srbiji. Ph.D. Thesis.

[40] Elham Azmoude M.S., Haniye Behnam B.S., Saeede Barati-Far B.S., Maryam Kabirian M.S. (2017). The Relationship of Socio-Demographic Factors, Fertility Behavior and Child’s Perceived Value with Fertility. Int. J. Commun. Based Nurs. Midwifery.

[41] Boboc C., Ghita S., Voinegau V. Quantitative analysis of the socio-demographic impact of family benefits. Proceedings of the 5th International Conference on Applied Statistics.

[42] Preda M., Mareci A., Tudoricu A., Taloș A.-M., Bogan E., Lequeux-Dincă A.I., Vijulie I. (2020). Defining the Concept of Family through the Lens of Fertile-Aged Women in Bucharest, Romania—Between Traditionalism and Inclusion. Sustainability.

[43] Peng R., Mou W., Xu P. (2023). Factors Associated with Fertility Intention among Chinese Married Youth during the COVID-19 Pandemic. Behav. Sci..

[44] Žvković J., Lukić T., Ivkov-Džigurski A., Đekić T. (2017). Policy responses to low fertility in Serbia. Case study of the municipality of Bela Palanka. Transylv. Rev. Adm. Sci..

[45] Hosseini M., Saikia U., Dasvarma G. (2021). The gap between desired and expected fertility among women in Iran: A case study of Tehran city. PLoS ONE.

